# Application of Remote Sensors in Mapping Rice Area and Forecasting Its Production: A Review

**DOI:** 10.3390/s150100769

**Published:** 2015-01-05

**Authors:** Mostafa K. Mosleh, Quazi K. Hassan, Ehsan H. Chowdhury

**Affiliations:** Department of Geomatics Engineering, Schulich School of Engineering, University of Calgary, 2500 University Dr NW, Calgary, Alberta T2N 1N4, Canada; E-Mails: mkkmosle@ucalgary.ca (M.K.M.); echowdhu@ucalgary.ca (E.H.C.)

**Keywords:** microwave remote sensing, optical remote sensing, rice acreage mapping, rice yield forecasting

## Abstract

Rice is one of the staple foods for more than three billion people worldwide. Rice paddies accounted for approximately 11.5% of the World's arable land area during 2012. Rice provided ∼19% of the global dietary energy in recent times and its annual average consumption *per capita* was ∼65 kg during 2010–2011. Therefore, rice area mapping and forecasting its production is important for food security, where demands often exceed production due to an ever increasing population. Timely and accurate estimation of rice areas and forecasting its production can provide invaluable information for governments, planners, and decision makers in formulating policies in regard to import/export in the event of shortfall and/or surplus. The aim of this paper was to review the applicability of the remote sensing-based imagery for rice area mapping and forecasting its production. Recent advances on the resolutions (*i.e.*, spectral, spatial, radiometric, and temporal) and availability of remote sensing imagery have allowed us timely collection of information on the growth and development stages of the rice crop. For elaborative understanding of the application of remote sensing sensors, following issues were described: the rice area mapping and forecasting its production using optical and microwave imagery, synergy between remote sensing-based methods and other developments, and their implications as an operational one. The overview of the studies to date indicated that remote sensing-based methods using optical and microwave imagery found to be encouraging. However, there were having some limitations, such as: (i) optical remote sensing imagery had relatively low spatial resolution led to inaccurate estimation of rice areas; and (ii) radar imagery would suffer from speckles, which potentially would degrade the quality of the images; and also the brightness of the backscatters were sensitive to the interacting surface. In addition, most of the methods used in forecasting rice yield were empirical in nature, so thus it would require further calibration and validation prior to implement over other geographical locations.

## 1. Introduction

Rice is one of the most important crop/food for more than three billion people (*i.e.*, approximately 50% of the World's population) [1]. During 2012, rice was cultivated on about 11.5% of the World's arable land; *i.e.*, ∼160.5 million hectares of land was under rice [2], while the total arable land was ∼1395 million hectares [3]. It is usually grown almost everywhere in the World, and its production in 2012 was ∼730 million tonnes [2]. However, most of the rice (*i.e.*, ∼88% of the World's total production in 2010 [4]) has been grown in Asia where ∼60% of the World's population lives [5]. Among the prominent rice-producing countries, the seven largest producers were China (197.22 million tonnes), India (120.62 million tonnes), Indonesia (66.41 million tonnes), Bangladesh (49.36 million tonnes), Viet Nam (39.99 million tonnes), Myanmar (33.20 million tonnes), and Thailand (31.56 million tonnes); which accounted together for ∼80.12% of the 2010 World production [4]. In recent times, rice has provided ∼19% of the global dietary energy [6] and its annual average consumption *per capita* during 2010–2011 was ∼65 kg [7].

Despite the large global cultivated rice area and growing rice production in many countries, the total demands often exceed the production. In addition, the global rice consumption is projected to be ∼873 million tonnes in 2030 [8]. In the recent decades, two major issues like population growth (in particular in the major rice producing/consuming countries) [5] and climate change put enormous pressure on the global food demand and its production [9,10]. Since problems with food security persist in many areas of the World [9], in particular in the heavy rice consuming regions, robust and reliable tools for mapping and early forecasting of rice production are thus critical. This is the case as reliable and timely estimates of rice crop areas and its production are essential for providing information for planners and decision makers to formulate policies in the case of shortfall or surplus.

It is interesting to mention that the most common and widely used methods for estimating rice cultivated areas are the use of agricultural statistical data acquired through field visits and interviewing the farmers. The methodology for mapping area under rice cultivation is basically done through annual/seasonal sample surveys based on a number of sample clusters that are constituted all over the country for measuring cultivated area during the crop growing season. Each cluster is visited many times and areas are recorded by the field staffs, checked, and then processed by regional statistical officers. Despite its invaluable ability for understanding historical trends in rice area, this method is extremely tedious, time-consuming, less precise, costly, inconsistent, and labour-intensive [11,12].

On the other hand, in terms of yield/production forecasting, it also depends upon the data collection technique from ground-based field visits that constituted sample surveys based on crop harvesting experiments. These yield surveys are extensive as plot yield data are collected through stratified multistage random sample techniques. From the data obtained in this way yields can be forecasted at the regional and national level [13]. However, such a technique has three major drawbacks: (i) it is time-consuming, subjective, and prone to significant discrepancies as a result of insufficient ground observations that cause poor crop production assessment [11,12]; (ii) the outcomes are usually made available to the government and public after several months of the harvesting of the crop, and thus not useful for food security purposes [14]; and (iii) it is costly, depending on the survey areas, e.g., a quarterly field survey for the entire Philippines required about CAD$50,000 [15]. Currently, the ground-based data collection method is in practice in various countries across the world and some of the responsible organizations include National Bureau of Statistics of China, Central Statistical Office of India, and Bangladesh Bureau of Statistics. These organizations collect data at the basic administrative level/unit and then aggregate at the district, region and country-levels [16–18].

In this context, remote sensing-based methods have already been proven as an effective alternative for mapping rice area [19–33] and forecasting rice production [14,15,34–37]. The benefits of remote sensing technology include: (i) spatial coverage over a large geographic area; (ii) availability during all seasons; (iii) relatively low cost, since some optical images are freely available (*i.e.*, MODIS, Landsat); although radar data are usually a bit costly (e.g., CAD$4000 per scene); (iv) efficient analysis; (v) they provide information in a timely manner; and (vi) they are capable of delineating detailed spatial distributions of areas under rice cultivation. In addition, remote sensing-based methods for forecasting rice production may help the governments, planners, and decision makers to formulate appropriate policies to: (i) quantify either how much to import in the event of shortfall or optionally to export in case of a surplus [14]; and (ii) purchase rice sooner at a relatively cheaper price as other rice producing countries do not have information about this upcoming need. For these methods, either optical remote sensing-based surface reflectance or microwave remote sensing-based backscattering is usually used in both mapping and forecasting processes. One of the most important issues is that regardless the employed method (*i.e.*, ground or remote sensing-based) the user requires fast, reliable (accurate), less costly, and least labour-intensive ways; and also forecasting should take place prior to harvesting of the crop.

**Figure 1.**
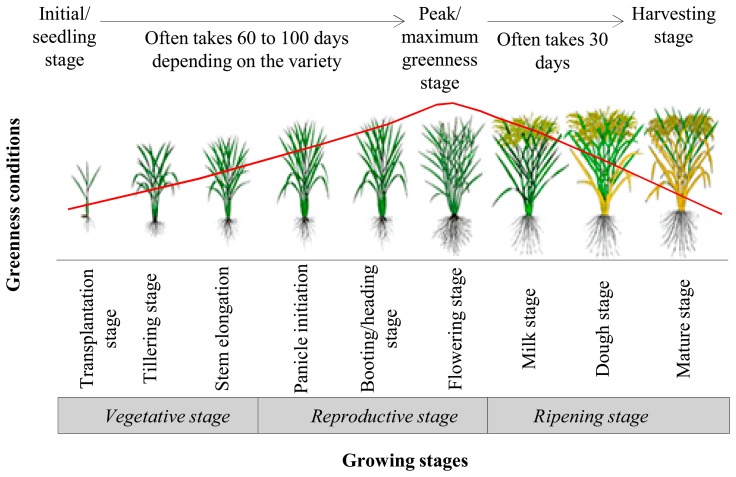
Growing stage of a typical rice crop and their associated greenness conditions (modified after [38]). The red curve shows a typical temporal dynamics of very commonly used remote sensing-based vegetation greenness index [*i.e.*, normalized difference vegetation index (NDVI)].

In this paper, our objective was to provide an overview of the use of remote sensing imagery for mapping rice crop areas and forecasting its production. The specific goals were to review three major issues, such as: (i) suitable remote sensing-based methods for mapping rice areas and their limitations; (ii) remote sensing-based methods for forecasting rice yield and their functional implications; and (iii) the synergy between remote sensing-based methods and other developments for mapping rice area and forecasting its yield. In case of mapping rice areas, remote sensing images acquired over either entire growing season or some of the critical stages (e.g., transplantation, tillering, heading/booting, flowering, *etc.*) were used, as shown in Figure 1. On the other hand, the images acquired during the peak/maximum greenness stage (see Figure 1) were commonly used in the event of forecasting rice yield. Figure 1 also shows that a remotely sensed vegetation index of normalized difference vegetation index (NDVI) would: (i) be low at the transplantation stage; (ii) increase over the vegetative-to-reproductive stage; and (iii) gradually decrease with the progression of the ripening stage.

## 2. Remote Sensing-Based Methods for Mapping Rice Area

Due to the ability of viewing the Earth's surface by the remote sensing platforms in a repetitive manner, several remote sensing-based methods were developed for mapping the rice areas in different parts of the World. Both optical and microwave remote sensing systems offer practical means for mapping rice areas which are described in the following sub-sections.

### 2.1. Optical Remote Sensing-Based Mapping Methods

Optical remote sensing sensors were widely used for mapping rice areas worldwide [19–33]. It was used in discriminating land use/land cover and measuring crop areas due to their ability to view the Earth surface in the spectral range 0.4 to 2.5 μm. The most commonly applied optical sensors include: Landsat (mainly MSS, TM and ETM+), SPOT-VGT, NOAA/AVHRR, MODIS, *etc*. These satellite sensors have the potential of obtaining multi-temporal and multi-spectral reflectance data over croplands that can be used for deriving time-series of vegetation indices (VIs), calculated as a function of red, blue, and infrared spectral bands (see the major VIs in Table 1). A number of studies have explored the usefulness of optical remote sensing sensors to identify rice areas; and some of the example cases are briefly described in Table 2.

### 2.2. Microwave Remote Sensing-Based Mapping Methods

One of the prime advantages of microwave remote sensing is associated with its ability of acquiring images theoretically under any weather conditions, such as cloud cover, rain, snow, and solar irradiance. Therefore, the radar images collected from microwave sensors provide an excellent imagery source for mapping rice areas, where rice cultivation takes place during rainy season with dominant cloudy conditions. Since the 1990s, researchers have explored the usefulness of microwave data retrieved from different satellites (e.g., ERS-1 and 2, RADARSAT-1 and 2, ENVISAT ASAR, *etc.*). In general, the temporal variation of radar backscatter over the growing season was the key factor in delineating rice areas; and some of the example studies are briefly described in Table 3.

### 2.3. Integration of Optical and Microwave Remote Sensing-Based Mapping Methods

In addition to the independent use of optical and microwave remote sensing-based methods, several studies have been conducted upon combining both of the methods together for mapping rice areas; and some of the example cases are briefly described in Table 4.

### 2.4. Limitations of Remote Sensing-Based Mapping Methods

For most of the optical remote sensing-based methods, the spatial resolution of the employed images (*i.e.*, MODIS, AVHRR, and SPOT-VGT) were relatively low (*i.e.*, in the range 500 m to 1.1 km). This particular issue would be critical as the dimension of some rice fields might be smaller than the spatial resolution of these satellite platforms. In the context of spatial resolution, the use of Landsat imagery would compensate the spatial resolution; however, sometimes it would be very difficult to obtain cloud-free images over some of the rice growing regions of the World. Also, temporal resolution (*i.e.*, 16 days) of the Landsat images and its swath coverage (*i.e.*, approximately 180 km) might restrict their application in rice mapping. These impose a problem for rice mapping especially when the period of interest falls in rainy season and during which heavy cloud greatly influences the image quality [21–23,58,78]. Another factor, such as variable topography would significantly impact the delineation of the rice areas as the surface reflectance from the hill terrain might be influenced by the adjacent areas [22,23,50,55]. In general, the microwave remote sensing platforms usually provide relatively higher spatial resolution images; however, several limitations exist, such as:

(i) It has an inherent problem of speckles, which look as a grainy “salt and pepper” texture in the image. These are formed due to random constructive and destructive interference from the multiple scattering returns that occur within each pixel/cell. These speckles degrade the quality of an image and make the interpretation process (visual or digital) more difficult [79].
(ii) The brightness of the radar backscatters are highly influenced by several factors, such as, volume scattering over vegetation, surface moisture conditions, surface roughness, local incidence angle, surface cover density, surface scattering properties, structure of the scattering surface, dielectric constant of the scattering material, and double bouncing from the right angle surface [80].
(iii) Geometric distortions, such as foreshortening, layover, and shadowing, exist in almost all radar imagery. For example, radar shadow occurs when the radar beam is not able to illuminate the ground surface [81]. Thus, all these factors have its influence during mapping rice cultivated areas, particularly in regions with steep topography and rough terrain.
(iv) Most of the studies of mapping rice area are based on single polarized image, where similar backscattering can occur from different land cover types. Hence, the multi-temporal dual polarization SAR imagery could be a promising source for rice mapping particularly in highly fragmented agricultural lands [66,82]. For example, Inou *et al.* [83] used a multifrequency (Ka, Ku, X, C and L) polarimetric (HH, VH, HV, and VV) scatterometer with four incident angles (25°, 35°, 45° and 55°) for the entire rice crop period, *i.e.*, before transplantation until postharvest cultivation in understanding the relation between backscattering and rice-related growth variables over Tsukuba, Japan. However, conducting a similar study like Inou *et al.* [83] would be quite expensive.
(v) Mapping rice area at large scale using radar data is costly, where the high cost of using continuous radar imagery making it inappropriate for seasonal rice crop monitoring and mapping.

## 3. Remote Sensing-Based Methods for Forecasting Rice Yield/Production

### 3.1. Optical Remote Sensing-Based Forecasting Methods

Researchers have dedicated significant efforts to forecasting rice yield using optical remote sensing images. The pre-harvest yield estimation could be possible as some of the spectral bands of the optical remote sensing satellites would be responsive to the vegetation conditions. For example, vegetation absorbs energy in the spectral range 0.45–0.70 μm, and reflects in the range 0.70–0.90 μm. By use of these spectral ranges, several vegetation indices have been employed, such as NDVI, RVI, DVI, IPVI, SAVI, vegetation condition index (VCI), vegetation health index (VHI), temperature condition index (TCI), and green normalized difference vegetation index (GNDVI) to estimate yield before harvesting and their applications are described in Table 5.

### 3.2. Microwave Remote Sensing-Based Forecasting Methods

Several studies have been conducted since 1990's for rice crop forecasting using microwave imagery and demonstrated encouraging results. Some relevant studies are described as follows:

- Shao *et al.* [66] used three RADARSAT-1 (C-band with HH polarization) images at different stages (*i.e.*, the end of the seedling period, flowering period, and beginning of the harvesting period) for rice yield estimation over Zhaoqing in Guangdong Province, Southern China. The temporal dynamics of the backscatter-values were investigated and found an accuracy of 91% compared with actual yield.
- Li *et al.* [88] used three RADARSAT-1 (C-band with HH polarization) images acquired during early, middle, and prior-to-harvesting stages for rice yield estimation over Guangdong Province, Southern China. A multi-regression model was developed between backscatter coefficients and yield (*i.e.*, rice biomass); and found a strong relation (*i.e.*, *r^2^* ≈ 0.91) with actual yield.
- Chen and McNairn [68] utilized RADARSAT-1 (C-band with HH polarization) for rice forecasting over Munoz and Santo Domingo, Philippine. They applied a neural network-based model to predict rice yield using the relationship between rice growth and radar backscatter; and found an encouraging prediction accuracy of 94% in comparison to actual yield.

### 3.3. Limitations of Remote Sensing-Based Forecasting Methods

It would be interesting to note that the limitations we discussed regarding the remote sensing-based methods for rice mapping (see Section 2.4) would also applicable for forecasting rice yield. In addition, the following factors would also be critical:

- All of the methods described above (*i.e.*, see Sections 3.1 and 3.2) were empirical in nature so that further calibration and validation would be required prior to implementing over other geographical locations. Technologically, empirical models were found to be relatively simple to build or develop, but these models could not take account of temporal changes in crop yields without long-term field experiments [89].
- These methods didn't describe the mechanism of delineating the rice acreage. Thus it might be possible to observe misclassification in case of mixing crop types [90].
- These methods were based on empirical/statistical models that needed to be performed on continuing basis and for different agro-climatic zones (*i.e.*, due to changing environmental conditions and weather shifts at different locations) [14,36].
- In general, a specific value of either optical remote sensing-based indices or microwave remote sensing-based backscattering coefficients might represent variable yield. This would be due to: (i) genetic variations; (ii) climatic conditions; (iii) soil types; and (iv) intra-species competitions. For example, Boro rice (which grows in winter) in Bangladesh demonstrated yields in the range of 1.93 to 4.69 metric ton per hectare during 2012 [91].

## 4. Synergy between Remote Sensing-Based Methods and Other Ones

Upon reviewing the existing literature, we found that the synergy between remote sensing and other methods could broadly be classified into two categories: synergy between remote sensing-based methods and (i) meteorological parameters; and (ii) crop growth models. These categories are briefly discussed in the following sub-sections.

### 4.1. Synergy between Remote Sensing-Based Methods and Meteorological Parameters

This approach is based on the integration of meteorological parameters with remote sensing-based methods for forecasting rice yield/production; and some example cases are summarized as follows:

- Prasad *et al.* [89] used AVHRR-derived 10-day composite of NDVI images integrating with meteorological parameters (*i.e.*, surface temperature, and rainfall) and soil moisture over India. A non-linear iterative multivariate optimization approach was used to derive an empirical piecewise linear crop yield prediction equation. The crop yield prediction model showed a high correlation coefficient that achieved accuracy greater than 90%.
- Sarma *et al.* [92] examined the importance of meteorological variables, *i.e.*, annual rainfall, southern oscillation index, sea surface temperature, growing degree day, combined with the AVHRR-derived NDVI in developing the statistical agro-climatic model in predicting the rice yield for kharif and rabi seasons over Andra Pradesh, India. The multi-regression model was employed; and found reasonable agreements between the actual and estimated rice yield (*i.e.*, *r*^2^ ∼ 0.71).
- Savin and Isaev [93] used a process-based model where the input variables included MODIS-derived 10-day composite of NDVI, fraction of absorbed radiation (fPAR), and meteorological variables (*i.e.*, temperature, and incident solar radiation) over the Republic of Kalmykia. During the period of maximum plant greenness, the model outcomes were the best (*i.e.*, always shown over prediction in the range 14%–48% in comparison to the actual yield).

### 4.2. Synergy between Remote Sensing-Based Methods and Crop Growth Model

The integration of remotely sensed data and rice crop growth models have become increasingly recognized as a potential tool for rice yield forecasting [94]. This approach relies on retrieval of biophysical crop parameters from remotely sensed data, which have been used as a direct input in rice crop models [95]. Various methods were found in the literature; some of such examples are as follows:

- Ribbes and Le Toan [96] used ERS-1 (C-band with VV polarization) and RADARSAT-1 (C-band with HH polarization) data to establish relationship between the backscattering coefficient and rice growth parameters, such as rice plant age, height, and biomass over Semarang and Jatisari, Indonesia. The sowing date and rice plant biomass values were determined from the relationships, and used as inputs in a rice crop growth model ORYZA1 (*i.e.*, simulates crop growth under irrigated conditions with optimum supply of nutrients, and without pest and disease infestation [97]) to estimate the rice yield. Good agreements were found between the simulated and no stress yield [*i.e.*, error below 15% (around 1.5 ton/ha) using RADARSAT; and 9% (around 0.7 ton/ha) using ERS data, respectively].
- Jing-Feng *et al.* (2002) [98] considered three types of AVHRR-derived NDVI-values, such as local area coverage (LAC)-NDVI, global area coverage (GAC)-NDVI, and radiometric measurements-NDVI; which were subsequently used as an input in the rice growth simulation model ORYZA1 [97] to develop a new model (called Rice-SAS model) to estimate rice yield for different growing seasons over Shaoxing province, China. The LAC-NDVI showed best estimates for rice yield with an estimation error of 1.03%, 0.79% and −0.79% for early, single, and late season, respectively.
- Shen *et al.* [99] used multi-temporal and multi-polarized ENVISAT ASAR (C-band with HH/VV polarization) data acquired during vegetative, reproductive, and ripening stages integrated with rice crop model ORYZA2000 (*i.e.*, simulates the growth and development of rice *in situ*ations of potential production, water, and nitrogen limitations [100]) for rice yield estimation over Xinghua, Jiangsu Province, China. The employed methods consisted of two steps: (i) generating a rice acreage map using ASAR dataset; and (ii) extracting the temporal dynamics of backscattering coefficients over the rice areas and assimilated with the ORYZA2000 model to predict rice yield. The obtained rice yield map was found to have over estimations by 13% on an average with a root mean square error of approximately 1133 kg/ha.
- Guo *et al.* [101] used multi-temporal MODIS data and HJ environment satellite-derived NDVI and EVI with LAI measurements integrated with rice production model ORYZA2000 [100] to estimate regional rice yield over Jiangsu Province, China. The obtained rice yield was found to have relative error of 6.31% compared with actual statistical data.

### 4.3. Limitations of Synergy between Remote Sensing-Based Methods and Other Ones

The synergy between remote sensing-based methods and other ones (*i.e.*, meteorological parameters, and crop growth models) provided promising results in both mapping and forecasting of rice crop. However, they have several limitations, such as:

(i) Utilization of meteorological data has several implications that include: spatial distribution of meteorological stations (*i.e.*, located sparsely in a large geographic landscape), incomplete and unavailable in a timely manner, and do not adequately represent the diversity over large areas [102,103];
(ii) Forecasting of rice production in the vicinity of the meteorological stations might be more accurate compared to other parts of the landscape, if the meteorological parameter-based models would be properly calibrated and validated. However, it would not be possible to install more and more meteorological stations across the landscape due to the expenses related to the installation, maintenance, data collection, and its processing [104];
(iii) Long-term meteorological data are hard to find in most of the rice crop growing countries in the world [35], which are inadequate for reliable forecasting of rice production;
(iv) In generating the spatial dynamics for a meteorological variable of interest, GIS-based interpolation techniques are usually used, which can produce different map outputs using the same input datasets [105]. Therefore, forecasting of rice production thus limits the use of meteorological based information over large geographic ex0tent; and
(v) Crop growth models are found to be more complex and require many input parameters. These include: several biophysical parameters (e.g., soil and meteorological variables) and plant parameters (e.g., biomass, LAI, and height, age *etc.*); which are usually expensive, labour-intensive, time consuming in acquisition [106].

## 5. Concluding Remarks

Here, we have provided an overview of using remote sensing sensors and their limitations in mapping the rice crop area and forecasting its production. In terms of the mapping the rice areas, the optical remote sensing imagery has relatively low spatial resolution, which results in over- and underestimation of the area under cultivation. The SAR imagery has several limitations in mapping rice areas, such as the fact speckles degrade the quality of the images, brightness of the backscatters is highly sensitive to the interacting surface, geometric distortions (particularly in irregular topography), low classification accuracy due to use of single polarization, and costly mapping of large geographic areas. In addition, the temporal resolution of microwave imagery was relatively low (*i.e.*, ranging between 24 to 44 days), which make it unfit for forecasting rice crop production prior to harvesting. Furthermore, the use of optical imagery in forecasting rice production was found to be encouraging, but mostly empirical-based. It is to be noted that most of the methods suffer from various aspects in particular how to implement them in other geographical locations *i.e.*, not transferable/extendable due to climatic variability (*i.e.*, temperature, precipitation amount, duration, and timing); soil characteristics (*i.e.*, texture, moisture capacity, *etc.*); management practices (*i.e.*, levee construction, fertilizer, irrigation, *etc.*); and selection of rice varieties (*i.e.*, tolerance to submergence, drought, salinity, disease and insect). Climate change is also evident in many regions of the World (*i.e.*, the average global temperature has increased by 0.74 °C in the last 100 years; rainfall has trended downward during 1960–2000 [107]) which will impact the rice crop productivity. Therefore, to assess and address the effect of climate change on the rice varieties (and its productivity), it is necessary to determine their genetic coefficients through carefully controlled experiments, trend of climate variation during the growth stages, and necessary to improve management practices to offset the adverse effects of climate change [108]. In addition, studies are required to evaluate the potential of applying multi-year remote sensing data for quantifying inter-annual variations of rice fields and its production due to extreme climate events (e.g., flooding, drought, cyclone, *etc.*) and/or human-driven land use changes. Also, factors such as plant diseases and insect infestation could be responsible for production losses, which could be monitored using remote sensing platforms [109]; however these were beyond the scope of this paper.

Despite the reasonable accuracies of rice area mapping and forecasting its production using remote sensing-based methods, such developments have many challenges as an operational one, which can be viewed as: (i) data acquisition; and (ii) development of appropriate methods. It is quite difficult to obtain cloud-free chronology of remote sensing data over the entire growing season of rice crop due to weather conditions (*i.e.*, humidity, cloud, and rainy conditions), which sometimes limit the availability and make acquisition problematic. Also, the availability of the satellite data must be assured. The development of image classification methods must be effective, efficient, and easy to implement. Based on the literature, upcoming satellites such as future MODIS and the RADARSAT constellation will enhance the temporal, spectral, and spatial resolutions that could be a good fit in mapping rice areas and forecasting its production.

## Figures and Tables

**Figure 2.**
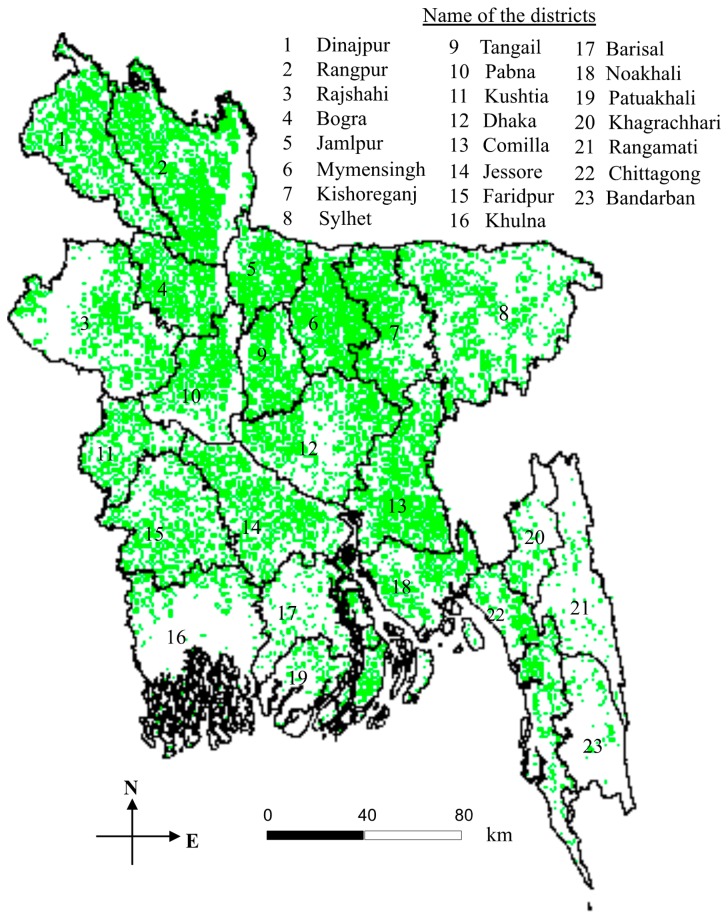
Example of MODIS-derived *boro* rice cultivation area (indicated using green shades) mapping during 2010.

**Table 1.** List of the common vegetation indices, and their mathematical formula, which have been used in mapping and yield/production forecasting.

**Index**	**Abbreviation**	**Formula**	**Reference**
Normalized Difference Vegetation Index	NDVI	$\frac{\rho_{\text{NIR}}-\rho_{R}}{\rho_{\text{NIR}}+\rho_{R}}$	[39]
Ratio Vegetation Index	RVI	$\frac{\rho_{\text{NIR}}}{\rho_{R}}$	[40]
Enhanced Vegetation Index	EVI	$2.5\times \frac{\rho_{\text{NIR}}-\rho_{R}}{\rho_{\text{NIR}}+6\times \rho_{R}+7.5\times \rho_{B}+1}$	[41]
Soil-Adjusted Vegetation Index	SAVI	$\frac{\rho_{\text{NIR}}-\rho_{R}}{\rho_{\text{NIR}}+\rho_{R}+L}(1+L)$	[42]
Land Surface Water Index	LSWI*	$\frac{\rho_{\text{NIR}}-\rho_{\text{SWIR}1}}{\rho_{\text{NIR}}+\rho_{\text{SWIR}1}}$	[22]
Normalized Difference Built-up Index	NDBI	$\frac{\rho_{\text{SWIR}1}-\rho_{\text{NIR}}}{\rho_{\text{SWIR}1}+\rho_{\text{NIR}}}$	[43]
Triangular Vegetation Index	TVI	$\sqrt{\frac{\rho_{\text{NIR}}-\rho_{R}}{\rho_{\text{NIR}}+\rho_{R}}}+0.5$	[44]
Difference Vegetation Index	DVI	*ρ_NIR_* − *ρ_R_*	[45]
Infrared Percentage Vegetation Index	IPVI	$\frac{\rho_{\text{NIR}}}{\rho_{\text{NIR}}+\rho_{R}}$	[46]
Perpendicular Vegetation Index	PVI	$\frac{\rho_{\text{NIR}}-\text{a}\times \rho_{R}-b}{\sqrt{1+a^{2}}}$	[47]
Rice Growth Vegetation Index	RGVI	$1-\frac{\rho_{B}-\rho_{R}}{\left(\rho_{\text{NIR}}+\rho_{\text{SWIR}1}+\rho_{\text{SWIR}2}\right)}$	[28]

Note: *ρ* is the surface reflectance values for blue (B), red (R), near infrared (NIR), Shortwave infrared (SWIR1 and SWIR2 are centered at ∼1.64 and 2.22 μm respectively); *L* = 0.5; a (gain) and b (offset) are derived from NIR *vs*. RED scatter plot. * In fact, Gao [48] developed the LSWI first, however the name was normalized difference water index (NDWI) using SWIR1 centered at 1.24 μm.

**Table 2.** Examples of optical remote sensing-based methods used for rice area mapping, which were usually evaluated against the agricultural statistical dataset unless stated differently.

**Sensor**	**Method**	**Outcomes**
Landsat MSS	Evaluated two classification schemas using four images (comprised of G, R, and two NIR spectral bands) acquired during the transplantation to canopy development stages. The first one was the use of maximum likelihood classifier for generating the rice maps. The second one was the use of a vector classifier using two nodes (*i.e.*, water and green canopy response).	Between the two schemas, the vector classifier was found to have better agreements (*i.e.*, ∼94%) during the calibration phase (*i.e.*, during 1983/84 season) over New South Wales, Australia. It was then applied during 1984/85 season and found similar agreements (*i.e.*, ∼95%) [49].
FORMOSAT-2	Used two images consisting of R, G, B, and NIR bands acquired during transplanting and tillering stages. Two classifiers were used: (i) geographic information system (GIS) object-based post classification (GOBPC); and (ii) pixel-based hybrid classification (*i.e.*, both unsupervised and supervised).	GOBPC was found superior than the pixel-based approach. The accuracy of rice mapping was found to be more than 94% over Yilan, Hualien, and Kaohsiung; and 82% in Yunlin, Taiwan [50].
Landsat TM	Employed one image comprising of R, G, and B bands acquired during the early growing season. Unsupervised classifier was used under two conditions: (i) cut the study area first then classify; and (ii) classify the entire image then cut the study area.	Between the two conditions, the later condition (classify and cut) demonstrated better accuracies of ∼81% for semi-late rice and 90% for early rice crop over Hubei, China [51].
	Carried out three procedures: (i) land use map and town boundaries were created; (ii) optimal combination of three bands (*i.e.*, B, NIR, and SWIR1) were selected based on optimum index factor using one image acquired during the booting stage; and (iii) then ISODATA, parallelepiped, and maximum likelihood classifiers were applied.	Parallelepiped classifier was found the best (*i.e.*, an accuracy of 82.85%) among all the employed classifiers over Longyou County, China [52].
Landsat ETM+	Implemented two masks: (i) desert area outside the irrigation boundary using an irrigation schema map; and (ii) cloudy area using supervised classifier of B, and thermal bands. They employed supervised classifier over two scenes (*i.e.*, one during the growing stage and the other during the harvesting stage) comprising of all spectral bands (except the thermal one) individually. They also fused the optical bands of the both scenes and then applied supervised classifier.	Observed better outcomes (*i.e.*, an accuracy of 98%) in case of using the fused images over Mali, West Africa [53].
	Used six images spanning from the plantation to the harvesting period. They evaluated relations between rice age and several vegetation indices such as NDVI, RVI, IPVI, DVI, TVI, SAVI and RGVI. Note that they introduced the concept of using RGVI.	Observed the best relation (*i.e.*, *r*^2^ of 0.90) existed between the rice age and RGVI during the calibration phase. The application of the model showed significant relations (*i.e.*, *r*^2^ of 0.97) over Bali, Indonesia [28].
Huan Jing (HJ-1A/B)	Deployed twenty seven images comprised of B, G, R, and NIR bands during the growing season of three rice types, such as early-, medium-, and late-season rice. In determining the pure rice pixels, they used support vector machine classifier. In addition, they used “rice area fraction index” in identifying the mixed pixels (*i.e.*, mix of rice with other crops).	Validated against Rapid Eye-derived rice maps and found accuracies of 99%, 99%, and 97% for early-, medium-, late-season rice, respectively over Hunan, China [54].
NOAA AVHRR	Employed NDVI images, *i.e.*, one image during the peak greenness stage in 1989; and four images during the vegetative to peak greenness stage in 1999. In both of the years, they implemented a density slicing approach for the area estimation.	Found over estimations, *i.e.*, ∼23.3 and 27.5% in 1989 and 1999 respectively for *boro* rice acreage over Bangladesh [55].
SPOT XS	Used three images comprising of G, R and NIR bands during the pre-flood and first half of the flood period (*i.e.*, the earlier stages of the rice season). They evaluated two classification approaches. The first one was the integration of supervised and unsupervised classification of a database formed by the principal component reduction. The second one was implementation of a series of steps, such as unsupervised classification, stratification, and supervised classification.	The second approach provided better accuracy (*i.e.*, ∼70%) over Niger Delta, Mali, West Africa [56].
SPOT VGT	Developed a “peak detector algorithm” to differentiate between rain-fed and irrigated rice crops. The 10-day composite NDVI images over three calendar years were used to determine cropping intensity (*i.e.*, number, timing, and peak values). Then the peak NDVI-values were lag-correlated with the long-term average rainfall regimes. They found a “single” peak NDVI for rain-fed rice; and “multiple” peak for the irrigated rice.	Found overall accuracy of 89% over Suphanburi, Thailand [57].
MODIS	Used forty six 8-day composite of three vegetation/wetness indices (that included LSWI, NDVI, and EVI) over the entire calendar year. The LSWI was in particular used to identify the initial period of flooding and transplantation of the rice; while NDVI and EVI was used for understanding greenness conditions of the crop.	Observed reasonable agreement (*i.e.*, *r*^2^ values ranging from 0.80–0.88) with Landsat ETM+ derived rice maps over Southern China [22].
	Employed ten 16-day composite of NDVI images over the entire growing season. The methods consisted of three steps, *i.e.*, (i) determining rice signatures using ISODATA clustering techniques; (ii) formulating a mathematical model for extracting rice areas on the basis of the signatures determined in the first step; and (iii) model calibration and its validation.	Observed reasonable agreements (*i.e.*, percentage error in the range −0.83 to 1.42% at country-level; and *r*^2^ in the range 0.69%–0.89% at district levels) over Bangladesh [58]; and an example *boro* rice acreage map of 2010 season is shown in Figure 2.
	Generated a potential rice cultivation area by digitizing a hardcopy land use map, and then used to mask two NDVI images acquired during early and late stage of rice plantation. Finally, maximum likelihood classifier was applied on the combined image for extracting the rice area.	Observed an overall accuracy of 95.7% over Zhejiang, China [59].
IRS LISS-III	Used two images per year comprising of G, R, and NIR acquired during the early and vegetative stages of rice. The employed methods consisted of: (i) maximum likelihood classifier; (ii) establishing relationship between classified image and GPS measured area; and (iii) estimation of the rice area under hill shades and non-visible area based on field survey.	Found a good relationship between: (i) classified image and GPS measured area (*i.e.*, *r*^2^ value of 0.91); and (ii) eye estimates and actual measurement (*i.e.*, *r*^2^ value of 0.95) within the buffer zone over Ri-Bhoi, Meghalaya, India [60].
	Utilized: (i) digital elevation model to calculate the slope classes and considered the classes between 0%–25% slopes; (ii) multi-date LISS and land use maps to identify rice cultivation areas; (iii) soil maps to extract suitable soils for rice crop. Finally all of the layers were overlaid to generate potential rice areas.	The use of LISS improved the assessment, *i.e.*, an additional 746.44 km^2^ potential rice areas were identified over Mizoram, India [61].

**Table 3.** Examples of microwave remote sensing-based methods used for rice area mapping, which were usually evaluated against the agricultural statistical dataset unless stated differently.

**Sensor**	**Method**	**Outcomes**
ERS-1 (C-band with VV polarization)	Implemented maximum likelihood classifier using four multi-temporal images acquired between 25–30 days of transplantation to the initiation of flowering stage (*i.e.*, 60–70 days).	Found accuracy of 90 and 91.5% over Howrah and Hughly districts, respectively in West Bengal, India [62].
	Applied maximum likelihood classifier along with principal component analysis over six multi-temporal images acquired during the entire growing season.	Obtained an accuracy of 90% in comparison with the land use survey and Landsat TM-derive maps over Akita, Japan [63].
ERS-2 (C-band with VV polarization)	Generated five change index (CI) maps from seven images acquired during the growing season. Then each pixel in these CI maps was classified into one of three classes: increasing, decreasing, or constant backscattering.	Compared against SPOT-derived rice maps and found 93.2% agreements over Mekong River Delta, Vietnam [64].
RADARSAT-1 (C-band with HH polarization)	Deployed three multi-temporal images for each of the standard and fine beam modes acquired during transplanting and reproductive stages.	Found strong relation with an accuracy of 87% when compared to the available land cover map over Java Island, Indonesia [65].
	Applied a neural network classifier and post classification filtering over three multi-temporal images acquired during early growth/transplanting, flowering, and harvest stages.	Observed accuracy of 97% over Zhaoqing and Guangdong, China [66].
	Implemented a knowledge-based decision rule classifier based on the temporal variations of SAR backscatter of all land-cover classes using three multi-temporal images acquired during transplanting, and vegetative stages.	Noted an accuracy of >98% over Baleshwar and Bhadrak districts, Orissa, India [67].
	Used nine and ten multi-temporal images acquired during dry and wet seasons respectively. They carried out four classification approaches (*i.e.*, neural network classification; maximum likelihood classification; change detection; and an integration of change detection and neural network).	Revealed that the integrated method performed well with an accuracy of >96% over Munoz and Santo Domingo, Philippine [68].
	Executed a combination of entropy decomposition and support vector machine methods using three multi-temporal images acquired during vegetative, reproductive/peak, and ripening stages.	Found an accuracy of 95.3% when compared to the maximum likelihood classifer-dervied maps over Sungai Burung, Selangor, Malaysia [69].
ENVISAT ASAR (C-band with HH/HV polarizations)	Developed empirical relationships between backscattering coefficient, height, and biomass of rice using four multi-temporal HH and HV polarized images.	Observed an accuracy of 81% over Southern China [70].
ENVISAT ASAR (C-band with VV and HH polarization)	Implemented image difference technique using three pairs of images acquired during flooding, reproductive/peak, and ripening stages for each of the VV and HH polarization.	Found the best results from the difference image of HH polarization (*i.e.*, producer's and user's accuracies were 94% and 87% respectively) over Fuzhou, China [71].
ALOS PALSAR (L-band with HH polarization)	Applied support vector machine classifier based on the temporal variation of the backscatter using three multi-temporal images acquired during transplanting, vegetative, and heading stages.	Obtained user's and producer's accuracies of 90% and 76% respectively over Zhejiang, southeast China [72].

**Table 4.** Examples of integrating the optical and microwave imagery remote sensing data in mapping rice areas; which were usually evaluated against the agricultural statistical dataset unless stated differently.

**Sensor**	**Method**	**Outcomes**
Landsat TM (visible and shortwave infrared bands) and JERS-1 SAR (L-band with HH polarization)	Applied unsupervised classification over TM image to determine arable land area during dry season; and used SAR data to delineate rice areas during rainy season.	Found the estimated rice areas were 12%–14% smaller over Indramayu, Indonesia [73].
IRS-1D LISS-III (G, R, and NIR bands) and RADARSAT-1 SAR (C-band with HH polarization)	Employed: (i) maximum likelihood classifier using LISS-III data acquired during dry and summer seasons to map dry-to-summer rice; and (ii) temporal analysis using SAR data to determine rainy season rice map. The outcomes were combined to produce map year-round rice.	Noticed agreements of about 96.6% for the year-round rice over West Bengal, India [74].
Landsat TM (visible and shortwave infrared bands) and RADARSAT-1 SAR (C-band with HH polarization)	Used three fusion algorithms (*i.e.*, principal component analysis, multiplicative, and Brovey) to merge TM acquired during growing period and three multi-temporal radar images acquired during early, vegetative, harvesting stages; and then applied three classification schemas (*i.e.*, maximum likelihood, Mahalanobis distance, and minimum to mean distance).	Observed that the Mahalanobis distance over the Brovey fused image provided the best results (*i.e.*, 87.41%) in comparison to the rice maps, that was produced through extensive ground truthing and TM images acquired earlier than the ones used in this study over Mazandarm, Iran [75].
Visible-to-shortwave infrared bands of MODIS and Landsat 7 ETM+; and ALOS PALSAR (L-band with HH polarization)	Employed both of the multi-temporal PALSAR and MODIS images to define rice phenology and inundation patterns; and then a single ETM+ image to characterise “lake/water bodies masking”.	Revealed a high overall accuracy of 89% over Poyang lake Watershed, China [76].
AWiFS (G, R, NIR, and SWIR1 bands) and RADARSAT-1 SAR (C-band with HH polarization)	Implemented hierarchical decision rule classification technique using: (i) two SAR images acquired during transplanting period; and (ii) AWiFS-derived NDVI, SWIR1/R and NIR/G ratios during the peak greenness stage.	Noticed that the deviation in the area calculated was 1.93 and −10.5% over Bargarh and Sonepur districts respectively in Orissa, India [77].

**Table 5.** Examples of optical remote sensing-based methods used for forecasting rice yield/production.

**Sensor**	**Method**	**Outcomes**
IRS LISS-1A	Used the ratio between NIR and R spectral bands derived from IRS LISS images in order to develop an empirical relationship with ground-based yield data.	Found the deviation of the estimated yield varied from 2% to 14%, with *r*^2^ of 0.52 and RMSE of 2.62 at the district level over Cuttack and Puri of Orissa, India [84].
MODIS	Used 8-day composite of NDVI values to determine NDVI_max_ at around 45–60 days since the plantation; and compared with the actual yield data.	Revealed strong relationship, *i.e.*, *r*^2^ of 0.89 over Tabanan Regency, Bali province, Indonesia [85].
	Utilized 8-day composite of EVI and leaf area index (LAI) during the heading stages. Eight models (*i.e.*, derived from linear, interaction, quadratic, and pure quadratic) of rice crop yield, EVI and LAI were developed.	Observed that the quadratic model based on EVI and LAI produced the best results during the ripening period for the spring-winter and autumn-summer rice crop, that is, *r*^2^ of 0.70 and 0.74, respectively over Mekong Delta, Vietnam [86].
Landsat ETM+	Established relations between NDVI-values at 63 days since the plantation and ground-based yield observation.	Found strong exponential relations (*i.e.*, *r*^2^ ≈ 0.85) with rice yield during model development phase. The application of the model revealed strong relations (*i.e.*, *r*^2^ ≈ 0.93) between ground-based estimate and the forecasted rice yield over Bali, Indonesia [87].
NOAA AVHRR	Used 7-day composite of NDVI and brightness temperature-values at 16 km resolution to calculate a set of vegetation health indices (*i.e.*, VCI, TCI, and VHI) in order to forecast yield for *Aus* and *Aman.*	For the *Aus*, both of indices VCI and VHI-values between the plantation and early growing season had similar relationship (*i.e.*, *r*^2^ ≈ 0.62) with the yield. In addition, comparison of ground-based and predicted rice yield was found to have *r*^2^ of 0.56. On the other hand, combination of VCI and TCI during the period of reproductive phase (*i.e.*, one/two months prior to harvesting) had strong relations (*i.e.*, *r*^2^ ≈ 0.97) for *Aman* yield. Also, the relationship between ground-based and predicted rice yield was found strong (*i.e.*, *r*^2^ ≥ 0.89) over Bangladesh [34,35].
SPOT-4	Employed various reflective spectral bands and their derivatives in the form of several vegetation indices (*i.e.*, red, near infrared, and vegetation indices of DVI, IPVI, RVI, NDVI and SAVI).	Showed strong relations (*i.e.*, *r*^2^ in between 0.75 to 0.89) with the yield at 90 days from sowing (*i.e.*, maximum vegetation growth stage). Further comparison between actual and predicted rice yield showed high correlation (*i.e.*, *r*^2^ in between 0.90 to 0.95) over Kafr El-Sheikh Governorate, Egypt [14].
